# Our Homes

**Published:** 1880-04

**Authors:** 


*Our Homes.* By Henry Hartshorne, A. M., M. D., formerly Professor of Hygiene in University of Pennsylvania, is another of the American Health Primers, published by Presley Blackiston, 1012 Walnut Street, Philadelphia, 1880.

It relates to the manner in which we should live, and thus escape abridging our lives and multiplying the "ills that flesh is heir to." by discussing the question " How shall we have Healthy Homes? The work commences with " Situation," and treats of "Construction," "Light," "Warmth," "Ventilation," "Water Supply," "Drainage," "Disinfection," "Population," and "Workingmen's Homes," the author contending that "we live too far from nature." The articles on ventilation and drainage are worthy of the attention of all housekeepers, and the treatise is in every respect a useful and interesting one, not surpassed by any of the series which have preceded it.

